# Multiple Myeloma-Derived Exosomes Regulate the Functions of Mesenchymal Stem Cells Partially via Modulating miR-21 and miR-146a

**DOI:** 10.1155/2017/9012152

**Published:** 2017-11-27

**Authors:** Qian Cheng, Xin Li, Jingru Liu, Qinmao Ye, Yanfang Chen, Sanqin Tan, Jing Liu

**Affiliations:** ^1^Department of Hematology, The Third Xiangya Hospital of Central South University, Changsha 40013, China; ^2^Department of Pharmacology & Toxicology, Boonshoft School of Medicine, Wright State University, Dayton, OH 45435, USA; ^3^Department of Histology and Embryology, Medical College, Hunan Normal University, Changsha 40013, China

## Abstract

Exosomes derived from cancer cells can affect various functions of mesenchymal stem cells (MSCs) via conveying microRNAs (miRs). miR-21 and miR-146a have been demonstrated to regulate MSC proliferation and transformation. Interleukin-6 (IL-6) secreted from transformed MSCs in turn favors the survival of multiple myeloma (MM) cells. However, the effects of MM exosomes on MSC functions remain largely unclear. In this study, we investigated the effects of OPM2 (a MM cell line) exosomes (OPM2-exo) on regulating the proliferation, cancer-associated fibroblast (CAF) transformation, and IL-6 secretion of MSCs and determined the role of miR-21 and miR-146a in these effects. We found that OPM2-exo harbored high levels of miR-21 and miR-146a and that OPM2-exo coculture significantly increased MSC proliferation with upregulation of miR-21 and miR-146a. Moreover, OPM2-exo induced CAF transformation of MSCs, which was evidenced by increased fibroblast-activated protein (FAP), *α*-smooth muscle actin (*α*-SMA), and stromal-derived factor 1 (SDF-1) expressions and IL-6 secretion. Inhibition of miR-21 or miR-146a reduced these effects of OPM2-exo on MSCs. In conclusion, MM could promote the proliferation, CAF transformation, and IL-6 secretion of MSCs partially through regulating miR21 and miR146a.

## 1. Introduction

Multiple myeloma (MM) is the second most common hematological malignancy and characterized by clonal proliferation of malignant plasma cells in the bone marrow (BM) [1]. Accumulating evidence indicates that MM cells can affect the function and phenotype of mesenchymal stem cells (MSCs), osteoclasts, and endothelial cells by releasing soluble factors such as cytokines/proteins [2] and extracellular particles [3], which in turn favor the progression of MM cells [4, 5]. For instance, MM cells can educate MSCs to acquire a tumor-like phenotype with the ability to secrete interleukin-6 (IL-6), IL-8, and TNF-*β*, which further promote MM survival [6, 7]. It has also been shown that cancer cells can affect the function and phenotype of MSCs through secreting soluble factors [8, 9].

Exosomes, through delivering biological molecules such as proteins and microRNAs (miRs), represent a novel component of tumor microenvironment and play an important role in the communication between cancer cells and MSCs [10]. Previous studies have demonstrated that exosomes released by cancer cells could be incorporated by MSCs and result in the cancer-associated fibroblast (CAF) transformation of MSCs [11–16]. These studies have shown that CAFs transformed from MSCs express fibroblast-activated protein (FAP), *α*-smooth muscle actin (*α*-SMA), and stromal-derived factor 1 (SDF-1) and display enhanced proliferation and secretion of cytokines including IL-6 and TGF-*β* which could contribute to a tumor-supportive microenvironment. Exosomes released by acute myeloid leukemia cells have been shown to promote MSC proliferation [12]. It has also been suggested that chronic lymphocytic leukemia-derived exosomes could induce CAF transformation and IL-6 secretion of MSCs through transferring exosomal miR-150 and miR-146a [14]. However, whether MM exosomes can regulate MSC transformation remains unclear.

Emerging evidence indicates that miRs could be responsible for the proliferation, CAF transformation, and cytokine secretion of MSCs [13, 15]. miR-21 is a well-known oncogenic miRNA during MM proliferation and invasion and also a critical regulator in CAF transformation of breast cancer [17, 18]. It has been reported that exosomes of leukemia cells carry high levels of miR-21 and regulate MSC functions [12]. miR-146a expression has been demonstrated to be associated with levels of IL-6 secretion in breast cancer [19]. Moreover, MSC overexpressing miR-146a resulted in an increased secretion of IL-6, which further supports MM survival [20]. However, the role of miR-21 and miR-146a in regulating MSC proliferation and transformation has not fully been understood.

In this study, we examined the effects of MM-derived exosomes on MSC proliferation, CAF transformation, and IL-6 secretion, as well as the role of miR-21 and miR-146a in these effects.

## 2. Methods

### 2.1. Cell Culture

Primary human bone marrow-derived MSCs from healthy individuals were purchased from Lonza (Basel, Switzerland). All the experiments were performed with cells maintained in culture until passage 6. MSCs were maintained in MSC Growth BulletKit™ Medium (Lonza) and supplemented with 10% FBS, 2 mM L-glutamine, and penicillin/streptomycin (final concentration: 100 units/ml penicillin and 100 *μ*g/ml streptomycin). MSCs were positive for CD105, CD166, CD29, and CD44 and negative for CD14, CD34, and CD45. Human MM cell lines OPM-2, RPMI 8226, and U266 were cultured in RPMI 1640 medium (Hyclone, USA), supplemented with 10% fetal bovine serum (Hyclone, USA), 2 mM L-glutamine, and antibiotics (Hyclone, USA).

### 2.2. Exosome Extraction and Purification

The extraction and purification procedures were performed according to the previous study with some modifications [21]. Briefly, OPM2 cells were conditioned in RPMI 1640 medium without FBS. When the OPM2 cells reached 80%–90% confluence, the supernatants containing exosomes were harvested. The exosomes were purified by the procedure of differential centrifugation and purification. In brief, the supernatants were centrifuged for 20 min at 2000*g* to remove cellular debris. The cell-free culture medium was centrifuged at 20,000*g* for 70 min and ultracentrifuged at 170,000*g* for 1.5 h to pellet exosomes. Exosome pellets were collected and diluted in filtered PBS. The collected exosomes were stored at −80°C and used for following experiments. The size and concentration of exosomes were analyzed by using Nano Tracking System Analysis (NTA) 300 (UK).

### 2.3. PKH26 Stain of OPM2 Exosomes

For exosome-uptaking experiment, purified exosomes derived from OPM2 (OPM2-exo) were stained using PKH26 membrane dye (Sigma, USA). Stained exosomes were washed in 2 ml of PBS, collected by ultracentrifugation as demonstrated above, and resuspended in filtered PBS. 10 *μ*g of the PKH26-stained exosomes or the same volume of the PKH26-PBS control was added and incubated for 24 h. The binding of OPM2-exo to the MSCs was observed with a fluorescence microscope (Germany). OPM2 cells were washed twice with PBS, stained with Hoechst 33342 for 5 min, and washed twice with PBS before being photographed.

### 2.4. Cell Proliferation Assay

Proliferation of MSCs was determined by various methods including MTT assay (Sigma, USA), Cell Counting Kit-8 (CCK-8, Dojindo, Japan) assay, and direct cell counting. For the MTT assay, MSCs were seeded at 1 × 10^3^ cells/plate in a 96-well plate and cocultured with 0 (PBS, vehicle control), 5, 10, 20, 40, or 80 *μ*g/ml OPM2-exo. After day 4, the cells were incubated with 20 *μ*l of 5 mg/ml MTT solution for 4 h at 37°C. After removing the medium containing MTT, 150 *μ*l dimethyl sulfoxide (DMSO) was added to each well to dissolve the formazan. The optical density (OD) was measured at 490 nm by using a microplate reader (BioTek, USA). We also conducted a more sensitive assay to evaluate MSC proliferation since it has been shown that the detection sensitivity of CCK-8 is higher than that of any other tetrazolium salts such as MTT, XTT, or MTS. Briefly, cells (1 × 10^3^ per well) were plated in 96-well plates in triplicate for culture (37°C and 5% CO_2_). In the following day, the cells were cocultured with the same concentrations of OPM2-exo in a final volume of 90 *μ*l for 4 days. After incubation, 10 *μ*l CCK-8 solution was added to each well and incubated for 2 h. Then, the absorbance at 450 nm was measured by the microplate reader (BioTek, USA). To directly count the cell number, MSCs were seeded at low density in 6-well plates (5 × 10^3^ cells/plate). After 24 h, OPM2 cells were washed 3 times with PBS and switched to serum-free media, and OPM2-exo (80 *μ*g/ml) was added. The medium was changed every 3 days and added with fresh OPM2-exo. The cell number was counted at days 1, 4, and 10 with the automated cell counter (Beckman, USA) after trypan blue staining.

### 2.5. CAF Transformation Assay

MSCs were seeded at 5 × 10^3^ cells/plate in 6-well plates. 12 h after seeding, MSCs were treated with OPM2-exo (80 *μ*g/ml) to trigger the CAF transformation. The medium was changed every 3 days and added with fresh OPM2-exo. After 10 days, cells and conditioned medium were then collected and prepared for the following analysis.

### 2.6. Quantitative Real-Time PCR Analysis (qRT-PCR)

RNA was treated with TRIzol (Invitrogen, USA). One microgram of RNA was transcribed to cDNA using TranScript cDNA Synthesis Kit (Takara, Japan), and qRT-PCR was performed using a Bio-Rad 96 System (Bio-Rad, USA) with SYBR Green II qPCR Premix (Takara, Japan). The primers were listed at Supplementary Material Table S1. The PCR was conducted at 95°C for 10 minutes, 50 cycles at 95°C for 30 seconds, 60°C for 30 seconds, and 72°C for 1 minute. We used GAPDH (FAP, *α*-SMA, and SDF-1) and U6 (miR-21, miR-146a) as the internal control for normalization and calculated the relative expression by the 2^−ΔΔCt^ method.

### 2.7. IL-6 ELISA Assay

The MSC-conditioned medium was centrifuged to remove cellular debris, and then, IL-6 protein concentrations were quantified by using the ELISA kit (Invitrogen, USA) according to the manufacturer's protocol. In brief, the conditioned medium of MSCs was harvested, and standard and sample extracts were added to the microplate precoated with an antibody specific for IL-6. HRP substrate was added to each well. The level of IL-6 was measured at 450 nm.

### 2.8. miR-21 and miR-146a Inhibition

2 × 10^5^ cell suspensions were seeded in 6-well plates and transfected with RNase-free water (vehicle (veh)), miR control (miRCtrl, 100 nM), miR-21 inhibitor, or miR-146a inhibitor by using DharmaFECT 1 Transfection Reagent (Dharmacon, USA) at days 1 and 6, separately. The transfection procedure was performed according to the manufacturer's instructions. Transfection efficacy was examined by qRT-PCR at day 10.

### 2.9. Statistical Analysis

Data were expressed as means ± SEM of three independent experiments. Statistical analysis was performed by using one- or two-way analysis of variance (ANOVA) (SPSS version 17.0, SPSS, USA). Differences were considered to be significant when *p* values were smaller than 0.05.

## 3. Results

### 3.1. miR-21 and miR-146a Were Rich in MM-Derived Exosomes and Their Levels in MSCs Were Increased after Coculture with OPM2-exo

**Figure 1:**
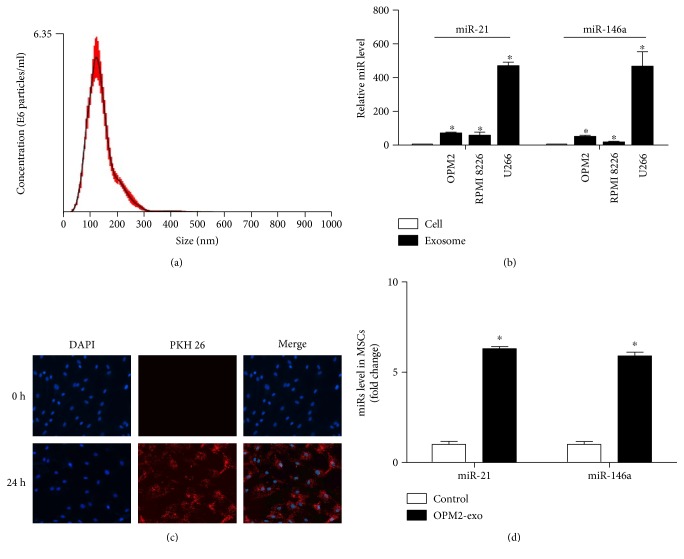
MM exosomes increased miR-21 and miR-146a levels in MSCs. (a) A representative picture of exosomes purified from OPM2 cells, examined by NTS300. (b) The expressions of miRs (miR-21, miR-146a) in parent MM cells (OPM2, RPMI-8226, and U266) and exosomes were measured by qPCR. (c) Fluorescence images of MSCs following the uptaking of OPM2-exo labeled with PKH26 (red) for 24 hours. (d) MSCs were cultured with OPM2-exo and the normalized expressions of miRs (miR-21, miR-146a) in MSCs were detected by qPCR. Results were shown as mean ± S.E.M. (^∗^*p* < 0.05, compared to untreated MSCs, *N* = 3/group).

As shown in Figure 1(a), NTA showed that the diameter of the isolated OPM2-exo was around 100 nm. qRT-PCR results demonstrated that exosomes derived from three MM cell lines (OPM2, RPMI 8226, and U266) contained higher levels of miR-21 and miR-146a when compared with those derived from parent MM cells (Figure 1(b)). OPM2-exo could be uptaken by MSCs after incubation for 24 hours analyzed by PKH26 stain (Figure 1(c)). With the treatment of OPM2-exo, we also observed the increased expressions of miR-21 and miR-146a in MSCs (Figure 1(d)).

### 3.2. OPM2-exo Promoted the Proliferation of MSCs in Dose- and Time-Dependent Manners

**Figure 2:**
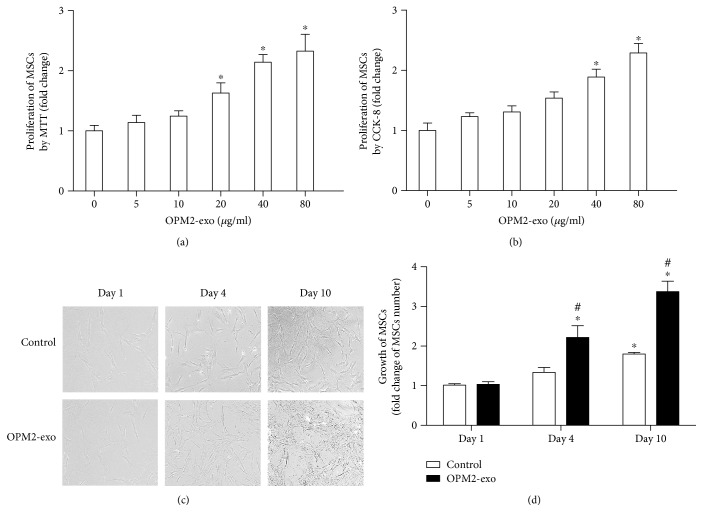
The effect of OPM2-exo on the proliferation of MSCs. (a) MSCs were treated with different concentrations of OPM2-exo for 4 days and proliferation was evaluated by using MTT assay. (b) MSCs were treated with different concentrations of OPM2-exo for 4 days and proliferation was evaluated by using CCK-8 assay. (c) Representative microscopy images of MSCs treated for 10 days with OPM2-exo (80 *μ*g/ml). 10x magnification in microscopy. Representative images of three independent experiments were reported at day 1, day 4, and day 10. (d) MSCs were treated with OPM2-exo (80 *μ*g/ml) and the cell number was counted by automated counter after day 1, day 4, and day 10, respectively. Results were shown as mean ± S.E.M. (^∗^*p* < 0.05, compared to untreated MSCs; ^#^*p* < 0.05, compared to day 1; *N* = 3/group).

We performed the proliferation assay by using different concentrations of OPM2-exo (0, 5, 10, 20, 40, and 80 *μ*g/ml) in coculture with MSCs. Both MTT (Figure 2(a)) and CCK-8 (Figure 2(b)) results showed that OPM2-exo promoted the proliferation in a dose-dependent manner. The optimal concentration for the OPM2-exo effect was considered to be 80 *μ*g/ml. Microscopy pictures showed that MSC displayed a clear increase in cell density in a time-dependent manner which is further enhanced with the coculture of OPM2-exo (Figure 2(c)). We next examined the effect of OPM2-exo on the growth of MSCs following treatment of OPM2-exo (80 *μ*g/ml) by directly counting the cell number. According to the cell count analysis, MSCs' number increased about 2 times after incubation with OPM2-exo at day 4 and over 2 times at day 10 (Figure 2(d)).

### 3.3. OPM2-exo Induced the Transformation of MSCs into CAFs with Increased IL-6 Secretion

**Figure 3:**
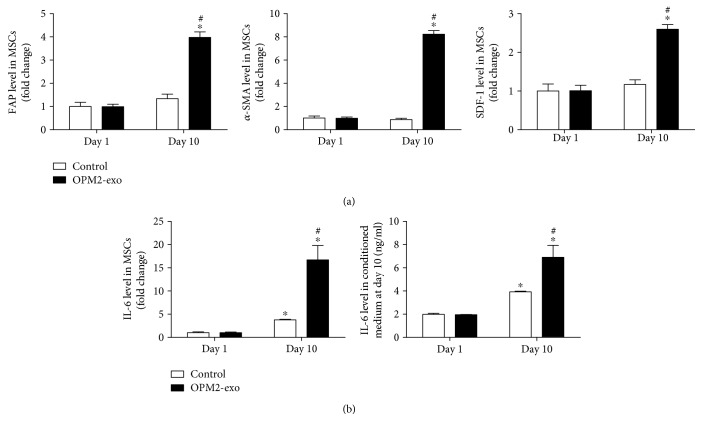
The effects of OPM2-exo on the CAF transformation and IL-6 secretion of MSCs. (a) Effect of OPM2-exo (80 *μ*g/ml) on the expressions of CAF transformation markers (FAP, *α*-SMA, and SDF-1) in MSCs at day 10. Data were expressed as normalized to GAPDH. Statistical analysis was performed by *t*-test. (b) Effect of OPM2-exo (80 *μ*g/ml) on IL-6 mRNA expression and IL-6 secretion of MSCs at day 10. Results were shown as mean ± S.E.M. (^∗^*p* < 0.05, compared to untreated MSCs; ^#^*p* < 0.05, compared to day 1; *N* = 3/group).

As noted in Figure 2(c), MSCs displayed a different phenotype cultured with OPM2-exo for 10 days, implicating the MSC transformation. We also examined the mRNA expressions of CAF transformation markers including FAP, *α*-SMA, and SDF-1. Results showed that OPM2-exo (80 *μ*g/ml) significantly induced the expressions of CAF transformation markers after being cocultured with OPM2-exo (Figure 3(a)). As shown in Figure 3(b), IL-6 mRNA was examined by using qRT-PCR and its level in the conditioned medium was measured by using ELISA after coculture with OPM2-exo (80 *μ*g/ml) for 10 days to observe the changes. The results showed that there was an increase in IL-6 mRNA expression as well as in its secretion of MSCs at day 10, which is significantly enhanced by the treatment of OPM2-exo (80 *μ*g/ml). Collectively, these results indicated that MSCs undergo CAF transformation in response to tumor exosome exposure.

### 3.4. Inhibition of miR-21 in MSCs Was Able to Inhibit the OPM2-exo-Induced MSC Proliferation and CAF Transformation

**Figure 4:**
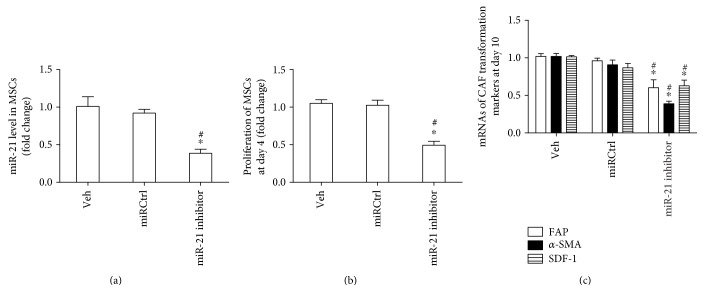
Inhibition of miR-21 decreased the proliferation and CAF transformation of MSCs. (a) MSCs were subjected to vehicle (veh) treatment and transfected with miRCtrl or miR-21 as indicated, and 10 days later, the miR-21 level was measured by qRT-PCR. (b) Effect of downregulating miR-21 on the proliferation of MSCs at day 4. (c) Effect of downregulating miR-21 on the expressions of CAF transformation markers in MSCs at day 10. Results were shown as mean ± S.E.M. (^∗^*p* < 0.05 versus veh; ^#^*p* < 0.05 versus miRCtrl, *N* = 3/group).

To elucidate the role of miR-21 in the proliferation and CAF transformation of MSCs, MSCs were transfected with miR-21 inhibitor and incubated with OPM2-exo (80 *μ*g/ml) for 10 days. The transfection efficiency of miR-21 inhibitor in MSCs was evaluated by qRT-PCR (Figure 4(a)). As expected, the level of miR-21 in MSCs was significantly decreased about 60% compared with that of veh or miRCtrl after transfection. Results showed that MSCs transfected with miR-21 inhibitor could significantly decrease the proliferation of MSCs when cultured with OPM2-exo for 4 days (Figure 4(b)). Additionally, inhibition of miR-21 decreased expressions of CAF markers including FAP, *α*-SMA, and SDF-1 in OPM2-treated MSCs at day 10 (Figure 4(c)).

### 3.5. Inhibition of miR-146a Could Reduce the IL-6 Expression and Secretion of OPM2-exo-Treated MSCs

**Figure 5:**
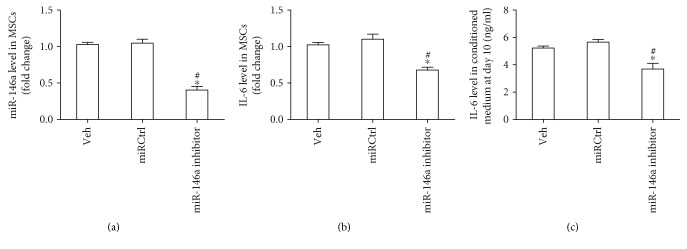
Inhibition of miR-146a decreased IL-6 secretion of MSCs. (a) MSCs were subjected to veh treatment and transfected with miRCtrl or miR-146a as indicated and 10 days later the miR-146a level was measured by qRT-PCR. (b) Effect of downregulating miR-146a on the IL-6 mRNA expression of MSCs at day 10. (c) Effect of downregulating miR-146a on the IL-6 secretion of MSCs at day 10. Results were shown as mean ± S.E.M. (^∗^*p* < 0.05 versus veh; ^#^*p* < 0.05 versus miRCtrl, *N* = 3/group).

To further elucidate the role of miR-146a in the IL-6 expression and secretion of transformed MSCs, MSCs were transfected with miR-146a inhibitor and cultured with OPM2-exo for 10 days. The transfection efficiency of miR-146a inhibitor was evaluated by qPCR (Figure 5(a)), and the results showed that the miR-146a expression was significantly inhibited about 60%. Inhibition of miR-146a was able to decrease the IL-6 expression and secretion of OPM2-exo-treated MSCs (Figures 5(b) and 5(c)).

## 4. Discussion

In the present study, we identified the effects of MM-derived exosomes on the proliferation, CAF transformation, and IL-6 secretion of MSCs, as well as defining the role of miR-21 and miR-146a in these effects.

Increasing evidence indicates that cancer exosomes could regulate the functions of MSCs probably through delivering their carried miRs [12, 14]. It has been reported that miR-21 and miR-146a play an important role in regulating MSC transformation and cytokine secretion [22, 23]. In this study, we analyzed that the levels of miR-21 and miR-146a in OPM2-exo and in MSCs after being coincubated with OPM2-exo. We found that miR-21 and miR-146a were enriched in OPM2-exo which enhanced the levels of these two miRs in coincubated MSCs. We also performed the qPCR analysis and found that miR-21 and miR-146a were significantly increased in exosomes from two other human MM cell lines (RPMI-8226 and U266). Our findings are consistent with previous reports showing that cancer exosomes can selectively package miRs which are able to be delivered into target cells for functioning [14, 24, 25]. For instance, exosomes of chronic lymphocytic leukemia have been shown to selectively deliver miR-21, miR-146a, miR-155, miR-148a, and let7-g to MSCs [14]. Our data suggest that miR-21 and miR-146a might be involved in regulating the functions of OPM2-exo on MSCs.

Previous studies have reported that exosomes derived from cancer cells could promote MSC proliferation [11, 12]. For instance, exosomes of T-cell leukemia/lymphoma cells are able to induce MSC proliferation, which is associated with the miR-21 expression [12]. Since miR-21 is selectively packaged in OPM2-exo, we further determined the effect of OPM2-exo on MSC proliferation and clarified whether miR-21 was the underlying mechanism. We found that MM exosomes were able to promote MSC proliferation in time- and dose-dependent manners. Moreover, we applied miR-21 inhibitor to further explore the role of miR-21 in OPM2-exo-induced MSC proliferation. Our results showed that miR-21 inhibitor significantly reduced the proliferation of MSCs induced by OPM2-exo. These data indicate that MM exosomes promote the proliferation of MSCs at least partly via miR-21, although the detail downstream pathway remains to be determined.

Exosomes derived from colorectal cancer, lung tumor, and leukemia have been shown to induce CAF transformation of MSCs [11–15]. CAFs are characterized by the expression of several markers including FAP, *α*-SMA, and SDF-1, as well as increasing secretion of cytokines [16, 26, 27]. The precise cellular origins of CAFs remain largely unclear; CAFs are reported to originate from various cell types such as resident fibroblasts [27], epithelial cells [28], and MSCs [8]. However, the role of MM exosome on CAF transformation of MSCs has not been determined yet. Our results showed that MSCs could be transformed to CAFs by OPM2-exos partially through the delivery of miR21 and miR146a and the activation of their downstream genes including IL-6, SDF-1, FAP, and *α*-SMA. It has been illustrated that miR-21 is highly associated with CAFs in breast and ovarian cancer [29, 30]. In this study, we also detected the association of elevated level of miR-21 with CAF transformation in OPM2-exo-treated MSCs. To confirm the role of miR-21 in CAF transformation of MSCs induced by OPM2-exo, we downregulated the level of miR-21 in MSCs by miR-21 inhibitor. Interestingly, we found that miR-21 inhibitor significantly decreased the effect of OPM2-exo on the expression of CAF markers, suggesting the involvement of miR-21 in CAF transformation of MSCs. Previous studies indicate that several genes including PTEN and PDCD4 are the downstreams of miR-21 in MSC. By inhibiting the expressions of PTEN, miR-21 could increase the levels of *α*-SMA, FAP, and SDF-1 in breast cancer. In this study, we found that miR-21 upregulated the expressions of *α*-SMA, FAP, and SDF-1 in the transformed MSC. Based on these, we tentatively attribute SDF-1, FAP, and *α*-SMA to be the target genes of miR-21. However, this hypothesis needs to be verified in the future.

Another important characteristic of transformed CAFs is their ability to secrete proinflammatory cytokines [13]. Frassanito et al. have reported that the CAFs of MM express high levels of TGF-*β* and IL-6 [16]. The cytokines secreted by CAFs, especially for IL-6, are believed to participate in the growth, angiogenesis, and metastases of MM [26]. Moreover, it is reported that IL-6 secretion of MSCs is regulated by miR-146a [20]. Since we detected that miR-146a was enriched in OPM2-exo and that the level of miR-146a in MSCs was increased after OPM2-exo coculture, we focused on IL-6 and miR-146a in this study. As expected, we found that IL-6 secretion was increased in transformed CAFs induced by OPM2-exo. Our data is supported by previous studies in gastric and lung cancer showing that cancer cell exosomes are able to elevate IL-6 secretion of transformed CAFs from MSCs [13, 15]. Moreover, we found that miR-146a inhibitor could significantly reduce the IL-6 expression as well as the IL-6 protein in the conditioned medium of OPM2-exo-treated MSCs. Our findings indicate that miR-146a is responsible for the increased IL-6 secretion of CAFs transformed from MSCs by MM exosomes. Previous studies have confirmed that miR-146a upregulates the expression of the Notch/IL-6 proinflammatory pathway; we postulate that IL-6 is the downstream gene of miR-146a in MSCs. Nevertheless, our deductions remain to be further elucidated.

## 5. Conclusions

In conclusion, our data have demonstrated that MM exosomes are able to promote the proliferation, CAF transformation, and IL-6 secretion of MSCs. Our results also suggest that miR-21 and miR-146a are involved in regulating the functions of MSCs. Our study highlights the important roles of MM exosomes and miRs in regulating the MSC functions and MM survival, which may be potential targets for MM therapies in the future.

## Supplementary Materials

Supplementary file The RT and qPCR primers used in this study.
