# Supplementary material for: Multiple Myeloma-Derived Exosomes Regulate the Functions of Mesenchymal Stem Cells Partially via Modulating miR-21 and miR-146a
Source: Stem Cells Int. 2017 Nov 27;2017:9012152. doi: 10.1155/2017/9012152 (PMC5733127; doi:10.1155/2017/9012152)
Supplement: Supplementary file — The RT and qPCR primers used in this study. [file 9012152.f1.docx]

Table 1: The RT and qPCR primers used in this study.

| Name |  | Sequence |
| --- | --- | --- |
| U6 | FR | TCGCTTCGGCAGCACATATAC |
|  | RR | GCGTGTCATCCTTGCGCAG |
|  | RT | CGCTTCACGAATTTGCGTGTC |
| miR-21 | FR | GTT GAC TGT TGA ATC TCA TGG CAA CA |
|  | RR | ATCCAGTGCAGGGTCCGAGG |
|  | RT | GTCGTATCCAGTGCAGGGTCCGAGGTATTCGCACTGGATACGACTGTCAG |
| miR-146a | FR | ACT GAA TTC CAT GGG TTG TGT CAG T |
|  | RR | ATCCAGTGCAGGGTCCGAGG |
|  | RT | GTCGTATCCAGTGCAGGGTCCGAGGTATTCGCACTGGATACGACACGATG |
| GAPDH | FR | ACCACAGTCCATGCCATCAC |
|  | RR | TCCACCACCCTGTTGCTGTA |
| FAP | FR | ATGAGCTTCCTCGTCCAATTCA |
|  | RR | AGACCACCAGAGAGCATATTTTG |
| α-SMA | FR | TCAATGTCCCAGCCATGTAT |
|  | RR | CAGCACGATGCCAGTTGT |
| SDF-1 | FR | ATTCTCAACACTCCAAACTGTGC |
|  | RR | ACTTTAGCTTCGGGTCAATGC |
| IL-6 | FR | CACCCCTGACCCAACCACAAAT |
|  | RR | TCCTTAAAGCTGCGCAGAATGAGA |
